# Simultaneous Chronic Invasive Fungal Infection and Tracheal Fungus Ball Mimicking Cancer in an Immunocompetent Patient

**DOI:** 10.1155/2016/2416452

**Published:** 2016-06-23

**Authors:** Erdoğan Çetinkaya, Mustafa Çörtük, Şule Gül, Ali Mert, Hilal Boyacı, Ertan Çam, H. Erhan Dincer

**Affiliations:** ^1^Yedikule Chest Diseases and Chest Surgery Education and Research Hospital, 34020 Istanbul, Turkey; ^2^Chest Diseases, Karabük University School of Medicine, 78200 Karabük, Turkey; ^3^Internal Medicine, İstanbul Medipol University School of Medicine, 34083 Istanbul, Turkey; ^4^Merzifon Kara Mustafa Paşa State Hospital, 05300 Amasya, Turkey; ^5^Pulmonary and Critical Care, University of Minnesota, Minneapolis, MN 55455, USA

## Abstract

Fungal infections of the lung are uncommon and mainly affect people with immune deficiency. There are crucial problems in the diagnosis and treatment of this condition. Invasive pulmonary aspergillosis and candidiasis are the most common opportunistic fungal infections.* Aspergillus* species (spp.) are saprophytes molds that exist in nature as spores and rarely cause disease in immunocompetent individuals. In patients with immune deficiency or chronic lung disease, such as cavitary lung disease or bronchiectasis,* Aspergillus* may cause a variety of aspergillosis infections. Here we present a case of a 57-year-old patient without immunodeficiency or chronic lung disease who was diagnosed with endotracheal fungus ball and chronic fungal infection, possibly due to* Aspergillus*. Bronchoscopic examination showed a paralyzed right vocal cord and vegetating mass that was yellow in color, at the posterior wall of tracheal lumen. After 3 months, both the parenchymal and tracheal lesions were completely resolved.

## 1. Introduction

Fungal diseases are often caused by fungi that are common in the environment. Fungal pneumonia is an infection of the lungs.* Aspergillus* species are a saprophyte fungus, and* Aspergillus* spores can reach the lungs with inhalation [1]. Although there are many subtypes of* Aspergillus*, few are pathogenic to humans.* Aspergillus fumigatus* is considered the most common pathogen [2].* Aspergillus *species do not usually cause disease in healthy individuals [1]. Various forms of* Aspergillus* infection can be seen in patients with suppressed immune system or chronic lung disease [3]. After inhalation of* Aspergillus* spores, aspergilloma can occur in patients with cavitary lung disease. Chronic fungal infection (CFI) may develop in patients with chronic lung disease or secondary immune deficiency, and invasive pulmonary aspergillosis (IPA) can develop in individuals with severe immunodeficiency and may also cause allergic bronchopulmonary aspergillosis (ABPA) in asthma sufferers [3].

Aspergilloma is defined as a fungus ball that is usually located in an existing cavity and does not invade the lung tissue [4]. Chronic pulmonary aspergillosis (CPA) is a syndrome that is usually seen in patients with underlying moderate chronic lung disease or immune deficiency and slowly destructs the lung tissue [5].

In this paper, we present a case without significant immunosuppression and chronic lung disease that was simultaneously diagnosed with an endotracheal fungus ball and CFA, possibly due to* Aspergillus*.

## 2. Case Report

A 57-year-old man with a productive cough resulting in yellowish sputum, fatigue, anorexia, and unintentional weight loss of approximately 18 pounds in 3 months presented to our clinic. In the last 15 days, hoarseness was added to his complaints. A *β*-lactam antibiotic had been administered to the patient during this time without improvement in his symptoms. His past medical history was significant for hypertension, type II diabetes mellitus, and he had a benign nodule located at his vocal cords and removed 6 years ago. He was taking an oral antidiabetic for diabetes and a calcium-channel blocker for hypertension. He was a former smoker who smoked during 10 pack-years and discontinued smoking 18 years ago. Physical examination revealed that right vocal cord was paralyzed, bilateral coarse rhonchi were observed, and lung auscultation was present more on the right side. Physical examination of chest revealed no stridor or wheezing. Results of other system examinations were normal. His blood pressure was 142/71 mmHg, pulse was 79 beats/minute, respiratory rate was 20 breaths/minute, and temperature was 99 degrees Fahrenheit.

**Figure 1:**
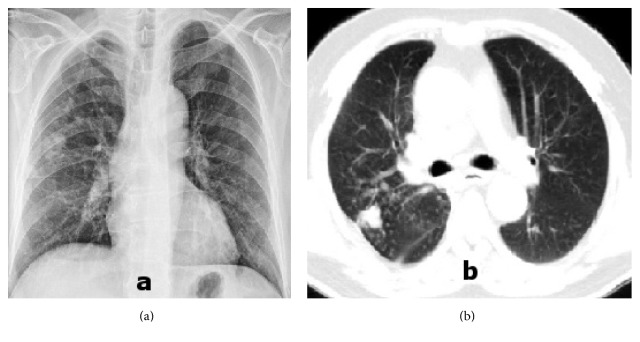
Pretreatment chest X-ray revealed a pulmonary nodule (a) and CT scan showed a nodule in the right upper lobe with tree-in-bud appearance in the adjacent parenchyma (b).

A chest X-ray showed a pulmonary nodule in the right mid lung area with a diameter of 15 mm and reticular infiltrate surround (Figure 1(a)). Computed tomography (CT) scans of the chest confirmed the presence of a nodule measuring 18 × 20 mm in the posterior segment of the right upper lobe with peribronchial thickening and a tree-in-bud appearance and in the adjacent parenchyma and pleural tenting (Figure 1(b)). A Ziehl-Neelsen stain for acidoresistant bacteria was negative in three sputum samples and negative in one bronchial lavage sample. Nonspecific bacterial, mycobacterial, yeast, and fungi cultures were also negative. Laboratory work showed a white blood cell count of 16500/mm^3^ (71.1% granulocytes), C-reactive protein of 14.7 mg/dL, sedimentation rate of 86, and normal glycated hemoglobin (HbA1c) levels. Pulmonary function tests showed that forced expiratory volume in 1 second (FEV1) was 1.62 L (56.9% of predicted), forced vital capacity (FVC) was 2.10 L (63.0% of predicted), and FEV1/FVC was 77.21. Human immunodeficiency virus antibody test was negative.

**Figure 2:**
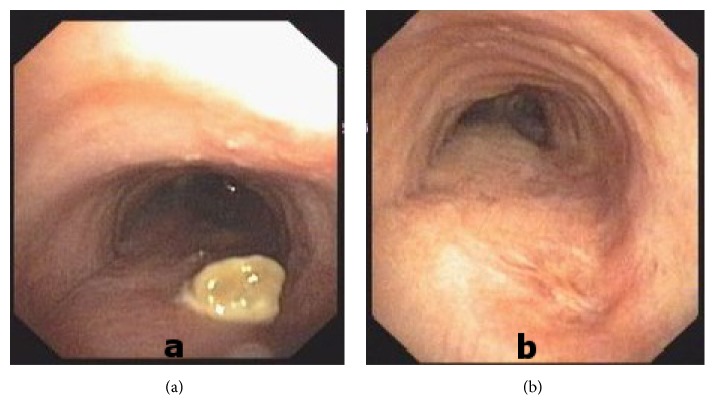
Bronchoscopic examination showed that the vegetating mass (a) is completely resolved after third month of treatment (b).

**Figure 3:**
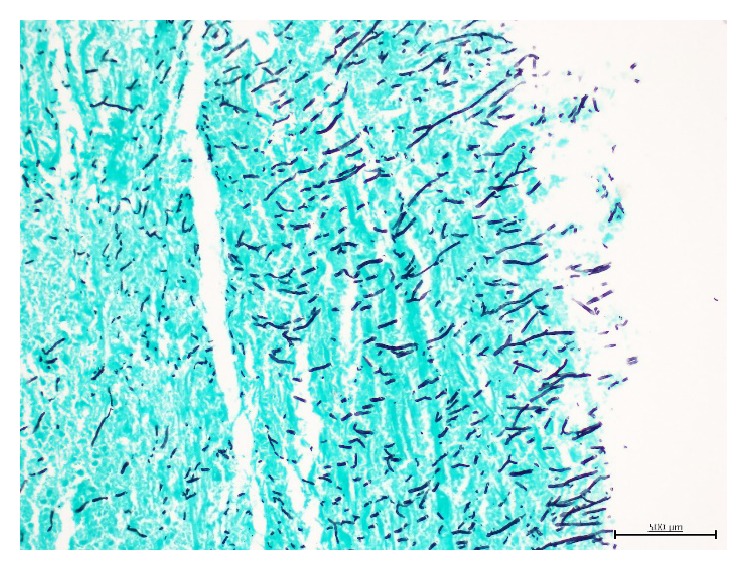
Grocott-Methenamine-Silver stain of biopsy of tracheal lesion showing acute angle hyphae with underlying necrosis (×200).

Bronchoscopic examination showed paralyzed right vocal cord and vegetating yellow color mass located 5 cm away from the vocal cords at the posterior wall of the tracheal lumen (Figure 2(a)). Biopsies, brush, and obtained bronchial lavage samples of the mass-like lesion revealed no malignancy; however, cytological evaluation showed fungal elements. Bronchoalveolar lavage and transbronchial forceps biopsies of the posterior segment of the right upper lobe lesion were stained positive using Grocott-Methenamine-Silver stain for hyphae with 45-degree branching, possibly due to* Aspergillus* on necrotic ground (Figure 3). Because we did not have the necessary equipment, we were not able to identify the specific type of fungi. The patient was started on oral itraconazole to treat the chronic fungal infection and the tracheal fungus ball. The treatment was completed at 3 months when both parenchymal and tracheal lesions were determined as being completely resolved by CT and bronchoscopic examinations (Figure 2(b)).

## 3. Discussions

*Aspergillus* species are filamentous fungi that commonly exist in nature. After being inhaled, if the host immunity is sufficient, it does not usually cause any disease. However, it can cause a number of diseases in patients with immunosuppression or with cavitary or chronic lung diseases [1]. Invasive pulmonary aspergillosis can be fatal, particularly in people with immunodeficiency. Chronic pulmonary aspergillosis is a disease seen in individuals who have moderately impaired immunity or impaired pulmonary defense mechanisms because of existing lung diseases [5]. Aspergilloma is a fungus ball that grows in a preexisting cavity and does not invade lung tissues. The fungus ball consists of hyphae, fibrin, and inflammatory cells [6]. Both CPA and endotracheal aspergillosis are uncommon [7]. Using a bronchoscope, an endotracheal aspergilloma has a yellow necrotic appearance. The macroscopic appearance of this view can be compared to malignity [8]. Although cases have been reported about endobronchial aspergilloma [9], the number of reported cases of endotracheal aspergilloma is considerably lower [10–12]. One of the presented cases had tuberculosis, one had chronic lymphocytic leukemia, and the other had lymphoma. Although diabetes mellitus may affect the cellular immunity, it rarely causes* Aspergillus* infection. Our case was a middle-aged man with no known medical problems except for well-controlled diabetes mellitus and hypertension. As far as we know, a case has not been published about CPA and endotracheal aspergilloma, which did not have significant immunosuppression.

Chronic pulmonary aspergillosis is an uncommon entity in immunocompetent individuals [1]. Because the symptoms are nonspecific, diagnosis may be difficult. Most have constitutional symptoms such as fever, weight loss, and a cough for at least three months. Demonstration of the fungus elements in the correct clinical setting and radiographic findings is necessary for a definitive diagnosis.

There is no consensus on the treatment of endobronchial aspergilloma. The preferred treatment is surgical excision. The use of antifungal therapy including voriconazole is recommended for chronic pulmonary aspergillosis [13]. Cucchetto et al. reported complete resolution in 42.9% of 21 patients treated with voriconazole [14]. In addition, it has been reported that itraconazole can be the treatment of choice [3]. In our case, oral itraconazole, used for 3 months, achieved complete resolution.

In summary, a fungus ball should be considered in the differential diagnosis when a necrotic lesion in endobronchial/endotracheal is found either in immunocompetent or in immunosuppressed patients. Chronic fungal infection may occur simultaneously. This case highlights the fact that CFI can be seen in patients with controlled diabetes mellitus in the absence of other immunosuppressed conditions.
